# Current advances in the molecular regulation of abiotic stress tolerance in sorghum *via* transcriptomic, proteomic, and metabolomic approaches

**DOI:** 10.3389/fpls.2023.1147328

**Published:** 2023-05-10

**Authors:** Min Tu, Canghao Du, Boju Yu, Guoli Wang, Yanbin Deng, Yuesheng Wang, Mingjie Chen, Junli Chang, Guangxiao Yang, Guangyuan He, Zhiyong Xiong, Yin Li

**Affiliations:** ^1^ School of Chemical and Environmental Engineering, Wuhan Polytechnic University, Wuhan, China; ^2^ The Genetic Engineering International Cooperation Base of Chinese Ministry of Science and Technology, The Key Laboratory of Molecular Biophysics of Chinese Ministry of Education, College of Life Science and Technology, Huazhong University of Science and Technology, Wuhan, China; ^3^ Laboratory of Forage and Endemic Crop Biology (Inner Mongolia University), Ministry of Education, School of Life Sciences, Hohhot, China

**Keywords:** sorghum, drought stress, salt and alkaline stress, temperature stress, omics analyses, gene expression regulation

## Abstract

Sorghum (*Sorghum bicolor* L. Moench), a monocot C4 crop, is an important staple crop for many countries in arid and semi-arid regions worldwide. Because sorghum has outstanding tolerance and adaptability to a variety of abiotic stresses, including drought, salt, and alkaline, and heavy metal stressors, it is valuable research material for better understanding the molecular mechanisms of stress tolerance in crops and for mining new genes for their genetic improvement of abiotic stress tolerance. Here, we compile recent progress achieved using physiological, transcriptome, proteome, and metabolome approaches; discuss the similarities and differences in how sorghum responds to differing stresses; and summarize the candidate genes involved in the process of responding to and regulating abiotic stresses. More importantly, we exemplify the differences between combined stresses and a single stress, emphasizing the necessity to strengthen future studies regarding the molecular responses and mechanisms of combined abiotic stresses, which has greater practical significance for food security. Our review lays a foundation for future functional studies of stress-tolerance-related genes and provides new insights into the molecular breeding of stress-tolerant sorghum genotypes, as well as listing a catalog of candidate genes for improving the stress tolerance for other key monocot crops, such as maize, rice, and sugarcane.

## Introduction

Globally, sorghum (*Sorghum bicolor* L.) ranks fifth in cereal crop production, serving as a staple for many countries in arid and semi-arid regions, being widely planted in tropical, subtropical to temperate regions as an important source of food, fiber, and fuel (Xie and Xu, 2019; Ngara et al., 2021). As one of the oldest cultivated cereal crops, the origin and domestication process of sorghum is very complex. To our best knowledge, the earliest evidence of human use of sorghum comes from Salt Lake Nabuta on the border between Egypt and Sudan, where carbonized sorghum grains were found and carbon-14 dated to 8000–8100 years old (Dahlberg et al., 1995). Sorghum cultivars may have originated from wild sorghum (*Sorghum verticilliflorum*) plants native to northeast Africa. The cultivation of sorghum ancestors may be traced back to 6 kyr before the present (kyr BP), and the domesticated bicolor race already existed ca. 5000 years ago in central eastern Sudan (Winchell et al., 2017; Smith et al., 2019).

As a C4 plant, sorghum features a combination of key traits: high photosynthetic efficiency, high nitrogen-use efficiency, outstanding tolerance to several abiotic stresses, and high biomass. These traits allow sorghum to be used not only as a food crop but also, more importantly, as a reliable feed resource and bioenergy crop (Hao et al., 2021). Sorghum cultivars can be grouped into grain sorghum, sweet sorghum, forage sorghum, and energy sorghum depending on their intended usage. Grain sorghum can serve as a staple or be used by breweries or as ingredient of pig feed, while forage sorghum cultivars attain high biomass and are used as dry forage. Interestingly, in sweet sorghum cultivars, the stem becomes a sink organ that accumulates a large amount of soluble sugars (*i.e*., sucrose, fructose, and glucose), making it suitable for the production of silage or bioethanol (Yu et al., 2008; Laopaiboon et al., 2009). For example, compare with the commonly used silage maize, some sweet sorghum varieties produce two to three times more biomass, which is ideal for silage use and holds promising potential for sustainable agriculture (Xie and Xu, 2019). Further, sorghum biomass may be used for the conversion of various industrial chemicals (Mathur et al., 2017).

Unlike the major C4 feed crop maize and the sugar crop sugarcane, sorghum can be planted on marginal lands due to its outstanding tolerance of a variety of abiotic stresses, including drought, high salinity, and low nutrition (Shan and Xu, 2009). Some sorghum accessions are distinguished by their strong absorption capacity for heavy metals and show potential in phytoremediation of soils contaminated by heavy metals (Feng et al., 2018). Accordingly, sweet sorghum has been proposed as way forward to simultaneously restore soil health and produce plant biomass, while the latter and its subsequently generated bioenergy are useful for our society but do not enter the food chain (Li, 2013; Jia et al., 2016). These prominent advantages of sorghum allow it to be considered as an environmentally- friendly green energy plant to utilize on marginal lands with low agricultural input, to diversify energy sources and relieve pressure on limited land resources for growing staple crops (Liu et al., 2007; Sticklen, 2007; Cai et al., 2011).

Contemporary advances in both energy sorghum and grain sorghum have been reviewed (Mullet et al., 2014; Boyles et al., 2019), and, more recently, the overall advantages and prospective applications of sorghum have been well discussed (Hao et al., 2021). Meanwhile, our understanding of the responses and adaptation of sorghum to various abiotic stresses has been substantially improved by the wide application of omic technologies. Due to the sessile growth, plants have evolved complex molecular networks to respond and survive in fluctuating environments; in turn, a variety of environmental factors negatively affect aspects of plants’ growth and development, leading to their yield declines and even death. These environmental stresses are generally divided into biotic versus abiotic, with the latter encompassing a wide range of factors, such as drought, salinity, alkalinity, high or low temperatures, and heavy metal toxicity. Among these abiotic stresses, drought, high salinity, and extreme temperatures are considered the most significant because they now severely threaten global food security and sustainable agriculture (Bailey-Serres et al., 2019). Extreme climatic events have adversely exacerbated global agricultural production in modern times (Fedoroff et al., 2010). It was recently estimated that, worldwide, drought conditions have caused about 30 billion dollars of losses in crop production (United Nations, 2011). Moreover, freshwater availability is predicted to drop by half due to climate change, far below than the expected global demand for agricultural water by 2050 (Gupta et al., 2020). Exacerbating matters, statistics have shown that about 6% of the global land area is damaged by salinization, yet effective measures are still lacking to control the spread of land salinization (Li et al., 2014). Collectively, this information highlights why and how abiotic stresses pose serious threats to agricultural production and food security, and that basic and applied research to mitigate the impacts of abiotic stresses deserves prioritization in most countries and global collaboration is imperative.

When facing abiotic stresses, plants utilize a combination of complicated biological and molecular processes, including morphological and physiological responses, tolerance, resistance, adaptations, and escapes. Usually, however, drought and salinity conditions induce multilevel stress signals (e.g., primary stress signals and secondary signals). Compared with primary signals, the secondary effects of drought and salt stresses are far more complex, including the induction of reactive oxygen species (ROS) stress, damage to biomolecules (such as membrane lipids, proteins, nucleic acids), and metabolic dysfunction (Zhu, 2016). The ability of plants to cope with and adapt to one or more given stresses after receiving corresponding stress signals is also generally referred to as stress tolerance (Zhang et al., 2022).

In the recent decade, numerous advances have been made in key genes regulating the response and adaptation to abiotic stresses in major crops (e.g., rice, maize, and wheat). It is known that crops can improve drought tolerance mainly by regulating root architecture and leaf transpiration efficiency, for instance, the *DEEPER ROOTING 1* gene (*DRO1*; Uga et al., 2013); Another example is that the receptor-like kinase *ERECTA* (*ER*) is major target to improve thermotolerance in rice (Shen et al., 2015). In addition, a cohort of drought-regulating transcription factors (TFs) including the members in the DREB, ERF, WRKY, ZFP, and MYB families have been functionally characterized in rice, wheat, and maize. For saline response and tolerance, the factors affecting salt tolerance mainly include the rate of water loss in leaves, water uptake capacity in roots, the ability to scavenge ROS and to maintain cell wall signaling perception and metabolism (Landi et al., 2017). Research on the mechanism of crop response to high temperature stress is mainly focused on the regulation of protein homeostasis and reactive oxygen species homeostasis. The response to low temperature stress mainly revolves around the CBF signaling pathway, whose components mainly including membrane localization proteins, protein kinases, calcium channels and E3 ubiquitin ligases play important roles in plant cold tolerance response (Wang L. et al., 2021).

It is generally accepted that field crops are often exposed to several stresses at the same time, yet most lab-based studies of the physiology and molecular mechanisms of stress responses and tolerance have focused upon a single stress condition or signal. For instance, drought conditions are often accompanied by raised temperature and/or high salinity, but combined stress conditions often cause more severe damage to the crop than does a single stress, in either an additive or synergistic way (Mittler, 2006; Barnabas et al., 2008). Therefore, the responses of plants to combined stresses and corresponding growth outcomes are usually difficult to predict from simply using data obtained from studies of a single stress factor, especially when stress conditions act antagonistically or lead to conflicting responses (Prasch and Sonnewald, 2015). One such example is drought and heat conditions in tandem. The heat stress incurred usually induces the stomata to open, to cool the leaves *via* transpiration; by contrast, stomata tend to close under drought conditions, to limit water loss. Work using Arabidopsis and tobacco has revealed that plants are unable to open their stomata under the combined stress of drought and heat, resulting in a higher leaf temperature (Rizhsky et al., 2002; Rizhsky et al., 2004). Generally, our understanding of plant stress biology is limited in two major ways: (1) effective responses and coordination vis-à-vis the combination of multiple abiotic stresses; and (2) transcriptional and metabolic responses and re-programming in a systems biology context.

In most plant stress-biology studies, the identification of key genes/alleles involved in stress tolerance and regulation from the species or genotypes exhibiting strong stress tolerance or adaptability is considered an efficient and effective approach. Hence, sorghum has been recognized as a target species for such gene identification purposes, given its higher tolerance to abiotic stresses compared to other major crops (e.g., rice, maize, and sugarcane). On one hand, gene functional studies of sorghum have lagged those of model crops (rice and maize), largely due to the difficulty in tissue culture, genetic transformation, and limited mutant resources (Grootboom et al., 2010). *Agrobacterium*-mediated transformation can be achieved in sorghum, but only in a few genotypes and with low efficiency (Hiei et al., 2014). On the other hand, there has been much effort directed to utilizing multi-omics technologies to decipher the systematic responses of sorghum to abiotic stresses and to identify key genes and signaling pathways involved in stress regulation and tolerance. Here, we aim to summarize recent achievements of sorghum stress tolerance based on multi-omic approaches (e.g., transcriptomics, proteomics and metabolomics), and to highlight the key genes in need of functional validation and of potential use in molecular breeding. Given the relatively abundant studies available in the literature, our review focuses on drought, salinity, and extreme temperature treatments, while studies on other abiotic stress conditions, for instance, nutrient deficiency (Gelli et al., 2017; Zhang et al., 2019b) and heavy metal toxicity (Feng et al., 2018; Jia et al., 2021), are out of the scope of the present review. Comparative genomics and population genomic approaches (e.g., quantitative trait loci mapping, and genome-wide association study, GWAS) have been powerful for unlocking the genetic architecture of complex traits in sorghum (reviewed in Hao et al., 2021). While there are some GWAS studies regarding abiotic stress tolerance, our present manuscript focuses on sorghum transcriptomics, proteomics and metabolomics (Chen et al., 2017; Chopra et al., 2017).

## Multi-omic research advances in drought and osmotic stresses

A sufficient water supply is one of the essential requirements for plant growth. Plants respond to water scarcity by altering their morphology, physiology, and biochemistry to mitigate the direct and indirect damage and to survive and/or to maintain their growth (Bray, 1993; Bray, 1997; Xiong and Zhu, 2002; Ngara et al., 2021). Plants’ responses to drought fall into three categories: drought escape, avoidance, and tolerance, which have been well defined in previous reviews (Farooq et al., 2009; Basu et al., 2016; Rodrigues et al., 2019).

Sorghum is generally recognized as a drought-tolerant crop. For example, the water consumption of sorghum in its full growth-period is 1.53 kg per plant whereas maize consumes 2.32 kg of water per plant (Su, 1995). The evapotranspiration of sorghum is 300~600 mm under field conditions, lower than that of maize (400~750 mm), rice (500~950 mm) or cotton (550~950 mm) (FAO, 1981). It is thought that sorghum plants are well tolerant to drought conditions not only because of the special cuticular wax metabolism but, more importantly, because of the coordination between root-to-shoot water use efficiency and a number of dynamic physiological adjustments to cope with drought conditions.

Dehydrins (DHN), a group of the late embryogenesis abundant (LEA) D-11 family proteins, accumulate in dehydrated plant tissues and may act as stabilizers of cell components (Close, 1996; Campbell and Close, 1997). Dehydrins could protect plants directly by scavenging ROS or providing an overall protective effect to those enzymes responsible for the dismutation of free radicals. For instance, *SbDhn1*- and *SbDhn2*-overexpressing transgenic tobacco plants are able to protect against oxidative damage (Halder et al., 2018). In addition, overexpressing *OsDhn1* in transgenic rice was shown to enhance their tolerance to drought and salt *via* ROS scavenging (Kumar et al., 2016). Wood and Goldsbrough (1997) found that the abundance of *SbDHN1* increased significantly in both seedlings and mature plants under a water deficit condition but were barely detectable in the well-watered and drought-recovered plants. A number of studies concluded that *DHN1* is drought inducible and may play an essential role in the plant response to drought stress (Close, 1996; Campbell and Close, 1997). The early and late responses of gene expression to the polyethylene glycol (PEG)-induced osmotic stress has been identified with microarray in the sorghum cultivar (cv.) BTx623, revealing ~2200 differentially expressed genes (DEGs) enriched in functions such as signaling transduction, gene expression regulation, dehydration protection, ROS scavenging, and defense (Buchanan et al., 2005). The PEG-upregulated genes include many encoding, for example, drought-responsive transcription factors and signaling proteins, LEA proteins, dehydrins, heat shock proteins (HSPs), ROS detoxification enzymes, biosynthesis of abscisic acid (ABA), and the metabolic enzymes of proline and raffinose family of oligosaccharides (RFOs). LEA proteins can enhance drought tolerance in many plants such as Arabidopsis, rice, wheat, and cabbage (Park et al., 2005; Xiao et al., 2007; Olvera-Carrillo et al., 2010; Chauhan and Khurana, 2011). Moreover, the proline and raffinose family of oligosaccharides (RFOs) plays a crucial role in how plants respond to drought stress. Overexpressing the galactose synthase gene *CsGolS4* in cucumbers led to significantly increased RFO content and drought resistance (Ma et al., 2021). Later, with the help of RNA-seq and a high-quality sorghum reference genome (Paterson et al., 2009), Dugas et al. (2011) uncovered changes in gene expression in response to the PEG or ABA treatment, finding that ABA could induce more genes than PEG, with the responsive genes (12% and 30% in the shoots and roots, respectively) differentially expressed in both treatments, suggesting ABA’s vital role in the osmotic response.

To further mine important genes associated with strong drought tolerance in sorghum, the up-and downregulated genes associated with drought stress were profiled in the drought-tolerant sorghum cv. XGL-1 with RNA-seq (Zhang et al., 2019a), uncovering that many differentially expressed genes in the roots were enriched in the functions such as sucrose metabolism and raffinose family oligosaccharide biosynthetic process. These results emphasized the importance to adjust carbohydrate metabolism during the response of roots to osmotic stress. Besides, Johnson et al. (2014) compared the gene expression disparities between the drought, heat, and drought and heat treatments, finding that only ~3.5% of the genes were drought-responsive and a small proportion (~20%) overlapped with previously detected osmotic responsive genes, underscoring the difference between drought and osmotic stresses (Johnson et al., 2014). Notably, the drought-upregulated genes include the proline biosynthetic gene (delta 1-pyrroline-5-carboxylate synthase 2, P5CS2-a; ID), the gene encoding sodium transporter (high-affinity K+ transporter 1, HKT1-a, Sb06g027900), LEA genes (Sb01g046490, Sb09g027110, Sb07g015410, Sb03g032380) and those related to lipid transport. Proline accumulation is a drought response conserved between plant species (Su et al., 2011). Arabidopsis pyrroline-5-carboxylate synthase 1 (AtP5CS1) catalyzes the first step in proline biosynthesis and is critical for proline accumulation under osmotic stress (Szekely et al., 2008). In addition, functional studies in sorghum revealed that SbHKT1;4 gene has selective uptake of sodium and potassium ions and is involved in regulating cellular ion homeostasis and improving tolerance to drought and salt stress (Wang et al., 2014).

More recently, several studies used the comparative approach to identify DEGs between stress-tolerant and stress-sensitive sorghum genotypes. A transcriptomic comparison of drought-sensitive and drought-tolerant genotypes (i.e., IS20351 and IS22330) indicated the former responded to the stress by hydrolyzing carbohydrates in roots, while the latter genotype gained tolerance by promoting the synthesis of anti-osmotic agents and antioxidants (e.g., proline, betaine, and glutathione; Fracasso et al., 2016). Also, the upregulation of lipid metabolic genes is associated with high drought tolerance in sorghum; for example, the phosphatidylinositol biosynthetic genes (Sb08g016610, Sb08g022520, and Sb05g026855) were upregulated in sorghum cv. IS20351. Some studies found that two sorghum genes (Sb06g014320 and Sb07g027910) encoding a glycerol phosphodiester phosphodiesterase and a monogalactosyl-diacylglycerol (MGDG) synthase, respectively, tend to be upregulated during drought stress but downregulated in drought-sensitive genotypes (Pasini et al., 2014; Fracasso et al., 2016). Similarly, transcriptomic comparison between two genotypes with contrasting drought tolerance during a post-anthesis drought treatment identified upregulation of genes related to antioxidant capacity and transmembrane transporters (Azzouz-Olden et al., 2020). Another comparative transcriptomic study discovered that drought induced more dramatic transcriptomic changes in roots than in leaves in terms of the number of DEGs and the extent of expression levels (Varoquaux et al., 2019). More importantly, a number of genes potentially conferring drought tolerance have been highlighted: (1) downregulation of several *WRKY* genes and jasmonic acid- and salicylic acid-responsive genes in roots indicates the possible balance between drought tolerance and inhibition of plant defense to microbes and pathogens (Pandey and Somssich, 2009); (2) downregulation of the key photosynthetic genes (e.g., the light-harvesting complex subunit B encoding gene, *LHCB*: Sobic.003G209800 and Sobic.003G209900) and their subsequent upregulation during drought recovery suggests photosynthesis could be the target for drought recovery; (3) upregulation of the proline biosynthetic gene P5CS2 (Sobic. 003G356000) points to proline accumulation as a common way to tolerate drought-induced damage (Funck et al., 2020); (4) the ROS-scavenging genes encoding glutathione S-transferase (*GST29*, Sobic.003G264400) was upregulated in both pre- and post- anthesis drought treatments and localized within a stay-green quantitative trait locus (the *Stg2* loci) (Thangaraj et al., 2022), suggesting a possible association between the drought-involved leaf stay-green trait and ROS scavenging ability (Harris et al., 2007).

Membranes are sensitive to drought stress and easily degraded and modified. Lipidomics and transcriptomics have been applied to profile membrane lipid dynamics in sorghum drought-sensitive cv. Hongyingzi and drought-tolerant cv. Kangsi (Xu et al., 2022). The unsaturation indices (UI) of dilauryl-diacylglycerol (DGDG), MGDG, phosphatidylglycerol (PG), and phosphatidylcholine (PC) all decreased in both cultivars under drought stress. By integrating transcriptomic and lipidomic data, some candidate genes regulating membrane lipids under drought stress were detected, namely CCT2(Sobic.001G282900), CER1 (Sobic.001G2222700), DGK1(Sobic.001G333900), DGK5(Sobic.003G318700), EMB3174(Sobic.001G403400), KCS4(Sobic.002G268500), LCB2(Sobic.003G412700), PAH1(Sobic.009G165400), PLDP1(Sobic.009G109900), PKP-β1(Sobic.003G244700), and KCS11(Sobic.010G181500).

Besides those genes identified, the changes in miRNA and proteome during drought stress have also been profiled in sorghum plants. Analysis of eight representative miRNAs among 11 sorghum genotypes phenotypically varied in drought tolerance revealed the drought-associated miRNAs, namely miR396, miR393, miR397-5p, miR166, miR167, and miR168 (Hamza et al., 2016). In particular, miR160, miR166, and miR396 targeted 28 transcription factors (TFs)- encoding genes including auxin response factor (ARF), homeobox-leucine zipper family protein (HD-ZIP), and growth regulating factors (GRF). Among these miRNAs, miR166, upregulated in sorghum, has been known to be related to drought tolerance in soybean (Kulcheski et al., 2011). In addition, members of the soybean HD-ZIPIII family, targeted by miR166, play a role in stress response to drought and salt stress conditions (Chen et al., 2014a). It has been recently found that a HD-ZIP TF *MdHB-7* enhanced the drought tolerance in apple by regulating ABA accumulation, stomatal closure, and ROS detoxification (Zhao et al., 2020). Collectively, these results indicate that the miR166-HD-ZIP module may also play an important role in drought stress regulation in sorghum. Nevertheless, it is worth noting that these miRNAs’ expression pattern during drought varies among sorghum genotypes, implying the complex involvement of miRNAs in drought-stress regulation and why further study to validate their functions in sorghum’s drought tolerance is needed.

Proteomics has also been employed to profile the protein dynamics of sorghum roots in response to PEG-induced osmotic stress (Li et al., 2020). During drought stress, the contents of MDA and proline, and the activities of superoxide dismutase (SOD), peroxidase (POD), and polyphenol oxidase (PPO), were gradually increased. Consistently, several antioxidant proteins (e.g., SOD, Sb07g023950, POD, Sb06g033850 and catalase, CAT, Sb10g030840), were upregulated as well, supporting the importance of ROS scavenger upregulation to cope with the stress. Another comparative proteomic study in sorghum focused on drought stress and recovery showed that: (1) the protein level of methionine synthase remained upregulated in the drought-tolerant sorghum lines but dropped in the drought-sensitive line; (2) the drought-sensitive and -tolerance sorghum lines exhibited contrasting changes in the cytosolic isoform of fructose-1,6-bisphosphate aldolase (FBA; Jedmowski et al., 2014). Similarly, the increase or maintenance of high levels of methionine synthase may be an osmoregulant metabolic approach to tolerate stress conditions (Merewitz et al., 2011). For the FBA-mediated carbohydrate metabolism, plastidic FBA indicates a disturbance of carbon fixation while cytosolic FBA may be active in the aldehyde detoxification process during stress. Moreover, Goche et al. (2020) employed the gel-free isobaric tags for relative and absolute quantitation (iTRAQ) proteomic technology to compare the root proteomes between a drought-sensitive and a drought-tolerant cultivar (ICSB338 and SA1441, respectively), thereby detecting drought-induced upregulation of proteins related to protein synthesis, proteasome inhibition, signaling transduction, and defense. Similarly, proteomic characterization of the osmotic-induced proteins in the BTx623 roots identified proteins related to protein synthesis, degradation, and defense (Li et al., 2020). Collectively, these proteomic studies in sorghum tend to emphasize that drought-tolerant cultivars tend to upregulate their stress-related signaling, protein synthesis and antioxidant activity to acquire better tolerance to drought conditions.

## Multi-omic research advances in salinity and alkaline stresses

Soil salinization is another major abiotic stress that severely limits crop production. About 6% of the global land area (~12 billion acres) is affected by salinization (Ismail and Horie, 2017), while the coverage of saline-alkali land has reached 100 million hectares in China. Moreover, secondary salinization of agricultural lands is becoming increasingly severe in China, posing a serious threat to the sustainable production of staple crops there (Fang et al., 2021; Wang L. et al., 2021). Generally, soil salinization refers to high salinity and alkaline stresses, which can distinctly influence plant growth. Salt stress is caused primarily by neutral salts (e.g., NaCl and Na_2_SO_4_). On one hand, high concentrations of sodium ions enter plant cells *via* ion channels and carrier proteins, resulting in ion toxicity. On the other hand, high concentrations of extracellular ions lead to greater extracellular osmotic potential, posing osmotic stresses and other secondary damage such as oxidative stress. By contrast, alkali stress is mainly caused by NaHCO_3_ and Na_2_CO_3_, either of which can raise the soil pH, which destabilizes the integrity of cell membranes and reduces root vigor in addition to ionic toxicity and osmotic damage (Zhang et al., 2017).

Many sorghum cultivars are capable of growing on salinized soils and even on marginal lands (Xie and Xu, 2019). To understand the molecular mechanisms of high salinity tolerance in sorghum, the cultivar M-81E was used as a representative salt-tolerant genotype for a series of transcriptome studies (Sui et al., 2015; Yang et al., 2017; Yang et al., 2018). In comparison to the salt-sensitive genotype Roma, M-81E harbors distinct groups of DEGs induced by salt. For example, genes related to photosynthesis are less influenced in M-81E: (1) *Lhca2-4* and *Lhcb6* encoding subunits in the light-harvesting complex are not inhibited in M-81E; (2) the genes encoding phosphoenolpyruvate carboxylase and pyruvate orthophosphate dikinase, respectively, remain unchanged in M-81E (Sui et al., 2015). Besides, sucrose synthase genes are upregulated in M-81E while the genes encoding sucrose catabolic enzymes are downregulated, and vice versa in Roma, patterns which suggest that maintaining photosynthesis and sucrose metabolism underpins the salt-tolerant sorghum. A more recent study revealed the roles of phytohormones in salt tolerance in sorghum: ABA and its signaling genes were increased in the leaves of the salt-tolerant cv. M-81E, while JA and its functionally related genes were upregulated in roots of the salt-sensitive cv. Roma (Yang et al., 2017). In addition, 2085 and 3172 DEGs were identified by RNA-seq in the roots of M-81E and Roma, respectively (Yang et al., 2018). In particular, many genes known to be involved in salt exclusion were found: (1) *SbHKT1;5* encoding a high-affinity potassium (K+) transporter (HAK) is dramatically upregulated in M-81E but only moderately increased in Roma; wheat *TaHAK1;5-D*, a homolog of *SbHKT1;5*, can enhance salt tolerance by expelling Na^+^ outside the cells (Byrt et al., 2014); and (2) the genes functioning in the exoplasmic barrier exhibit significant upregulation in M-81E. Altogether, these analyses suggest that enhanced potassium transportation and salt exclusion may explain the salt tolerance of M-81E. Another study just revealed the physiological and transcriptomic differences induced by drought and salt, respectively, highlighting that salt conditions significantly inhibit antioxidant enzymes and the auxin and cytokinin contents, while drought stress mainly impairs sugar metabolism in leaves (Wang et al., 2022).

Other omics analyses have further revealed additional layers of complexity in the salt responses of sorghum. Long non-coding RNAs (lncRNAs) regulate gene expression to modulate plant development and stress responses (Chen et al., 2020). Full-length transcriptomic comparison between the cultivars M-81E and Roma identified three upregulated lncRNAs (*lncRNA13472*, *lncRNA11310*, and *lncRNA2846*) in M-81E and two downregulated lncRNAs (*lncRNA26929* and *lncRNA14798*) in Roma (Sun et al., 2020). Functional predictions indicate that these salt-responsive lncRNAs might serve as endogenous RNAs (ceRNAs) acting to regulate the target genes related to ion transport, protein modification, transcriptional regulation, and synthetic transport of substances. A recent lipidomics analysis profiled changes to leaf membrane lipids during salt stress, finding a salt-induced decrease in both MGDG and PG (Ge et al., 2022). This result provides new insight into salt-induced membrane lipid remodeling in sorghum leaves and how that impacts the fluidity, stability, and integrity of their photosynthetic membrane system.

A proteomic study using the two-dimensional gel electrophoresis and mass spectrometry technique profiled differential proteins in sorghum leaves responsive to salt stress (Swami et al., 2011) and identified upregulated protein, including the universal stress protein (XP 002443333), glutathione S-transferase (XP 002458541, XP 002465442), peroxidase (XP 002463451, XP 002463451, XP 002463451) that are involved in the detoxification of reactive electrophilic compounds. Despite efforts made to understand the physiological and molecular aspects of sorghum’s response to and regulation of salt stress, such studies addressing the combination of salt and alkaline stresses remain quite limited. By using two-dimensional electrophoresis and proteomics, Dai et al. (2017) distinguished 30 upregulated proteins and 14 downregulated proteins under soda saline-alkali stress conditions (NaHCO_3_ and Na_2_CO_3_), functionally enriched in carbon fixation, carbon metabolism and glycolysis.

Metabolomics has also been employed to gain insights into the distinct salt tolerant phenotypes of sorghum genotypes (de Oliveira et al., 2020). (1) Significant changes of osmolytes (e.g., proline and soluble sugars) were detected in sorghum under salt conditions. Salinity triggered a pronounced increase in proline by 35% and 126% in CSF18 (salt-sensitive genotype) and CSF20 (salt-tolerant genotype), respectively. (2) Salinity induced an increase in putrescine in the salt-sensitive genotype but spermidine, spermidine and cadaverine were increased in the salt-tolerant genotype, suggesting that osmolytes and polyamines may play a role in conferring salt tolerance to sorghum. More recently, another metabolomics study in sorghum indicated that salt stress affects photosynthesis by repressing both chlorophyll and carotenoid metabolism, while sorghum mitigates salinity damage by inhibiting oxidative stress and increasing antioxidant content/enzyme activities (Punia et al., 2020). Further, the involvement of ion transport/signaling-related genes (SOS1 [XM_015763865.2], SOS2 [KP330207.1], NHX-2 [EU482408.2], V-PPase-11 [GQ469975.1], CIPK24 [XM_002438609.2], PP2A [XM_002448914.2]) in salt stress regulation was verified in sorghum by qPCR-based expression analysis (Ma et al., 2020). A recent study integrated transcriptome and metabolome analyses discovered the dynamic changes in flavonoid metabolic pathways from moderate and severe saline−alkali stress. That is, (1) flavonoid synthesis—particularly of naringenin, chalcone, prunin, naringin, and some kaempferol derivatives—is significantly upregulated under the moderate saline−alkali stress but repressed under the severe saline-alkali stress; and (2) cyanidin is specifically accumulated under the severe saline−alkali stress, which might protect cells from severe oxidative damage. Coupling the transcriptomic and metabolomic data identified several stress-induced flavonoid metabolic genes, including the naringenin-correlated flavonoid 3’-hydroxylase (*F3H*) genes (Sobic.004G201100, Sobic.004G328700, and Sobic.006G254000), a shikimate O-hydroxycinnamoyl transferase (*HCT*) gene (Sobic.006G136900), and a chalcone-flavanone isomerase (*CHI*) gene (Sobic.001G035600). Evidently, the application of metabolomics is providing novel insight into the role of flavonoid metabolism in sorghum’s salt tolerance.

## Multi-omic research advances in high- and low-temperature stresses

In recent years, the climate is fluctuating more widely, and extreme weather disasters are now occurring more frequently. Thus, extreme temperature stresses have emerged as a major threat adversely affecting plant growth and crop production (Farooq et al., 2011).

Raised temperature (also known as heat stress) is particularly harmful when it happens during the reproductive stage of plants, because heat stress inhibits metabolism, causes chloroplast oxidative damage, affects reproductive organ development and suppresses vital nutrient accumulation in seeds. During heat stress, stress-induced accumulation of misfolded proteins can be sensed by heat shock proteins (HSPs) and further activate heat stress transcription factors (HSFs) and their downstream heat stress-responsive target genes (Scharf et al., 2012; Zhang et al., 2022).

A recent quantitative proteomic study investigated the heat-responsive proteins that were secreted into the extracellular matrix (Ngcala et al., 2020), revealing 31 secreted, heat-responsive proteins that falls into the classical secretory pathways including metabolism, detoxification, and protein modification. This proteomic study provides a useful, timely resource of extracellular proteins that could serve as targets for developing heat-tolerant crops. Moreover, some genes such as the *leucine-rich repeat* (Sobic.005G126200), *cysteine proteinase inhibitors* (Sobic.003G126800 and Sobic.001G324800), and a *glycosyl hydrolase* (Sobic.002G055700) were validated by qPCR as heat-induced proteins.

By contrast, cold stress negatively impacts plant growth through distinct physiological mechanisms. First, cold stress decreases stomatal conductance and mainly affects photosystem II to reduce the net photosynthetic rate (Guo et al., 2021). Second, cold stress leads cell membranes to change from a liquid crystal phase to a gel phase, thereby impairing membrane permeability, and can even fully disrupt it (Chen et al., 2014b). Third, cold stress causes ROS bursts in cells. Plants respond to cold stress *via* C-repeat binding factors (CBFs)/dehydration-responsive-element binding factors (DREBs) for reprograming the metabolism (e.g., sugar and amino acid metabolism) to temporally adjust to the stress (Zhao et al., 2015; Ding et al., 2019; Guo et al., 2021).

Since sorghum originated in tropical and subtropical regions, most of its varieties are sensitive to low temperatures, especially during their germination and seedlings stages (Fiedler et al., 2016). So, to better understand the response to cold stress and to identify genes for improving cold tolerance in sorghum, several studies employing omics approaches have since been carried out. Transcriptomic comparison of seedlings of the cold-sensitive cv. BTx623 vis-à-vis those of the cold-tolerant cv. Hongkezi (HKZ) found several TF-encoding genes—i.e., *DREBs, CBFs*, and *ethylene responsive factors* (*ERFs*)—were drastically induced by the cold stress (Chopra et al., 2015). Additionally, several members from the plant cytochrome, glutathione s-transferase, and heat shock protein families were differentially regulated by cold treatment between those two cultivars. Nuclear factors Y (NF-Ys), these consisting of three subfamilies (i.e., NF-YA, NF-YB, and NF-YC), are involved in how plants respond to various stresses through complex interactions to form different hetero-trimmers (Petroni et al., 2012). Genome-wide characterization of the sorghum NF-Y family and expression analysis has revealed many members responsive to cold or heat stresses: For example, (1) *NF-YA2/4/6/7/8*, *NF-YB2/7/10/11/12/14/16/17*, and *NF-YC4/6/12/13* are induced by heat stress (40°C), and some of these genes are also regulated by cold stress (4°C); (2) *NF-YA8* is induced by both cold and heat stresses; (3) stress-related cis-elements, ABA-responsive element (ABRE), and heat shock-responsive element (HSE), are found in the promoter regions, but not the drought-responsive elements DRE and MYB (Maheshwari et al., 2019). Comparing the cold-induced DEGs between the cold-tolerant cv. Hongke4 and the cold-sensitive cv. SC407 uncovered not only classic cold-inducible genes—e.g., *CBL-interacting serine/threonine-protein kinase* (*CIPK16*; Sb02g024770) and *stress-activated protein kinase-3* (*SAPK3*; Sb01g028760)—but also important TFs warranting further functional studies (*MYB62*; Sb04g026210, *NAC1*; Sb01g003710, *WRKY55*; Sb02g011050, *WRKY51*; Sb03g003360 and *WRKY33*; Sb03g038510) (Jin et al., 2021; Zhou et al., 2022b). These cold-induced genes enriched metabolic functions, namely phenylpropanoid synthesis, carbon metabolism, amino acid biosynthesis, and starch and sucrose metabolism, thus indicating extensive metabolic reprogramming occurs under cold stress. Another transcriptomic comparison between the cold-tolerant cv. P61 and the cold-sensitive cv. H21 emphasized the differences in photosynthesis inhibition and oxidative damage (Shao et al., 2021). For example, the cold-tolerant cultivar showed significant upregulation for eight *SbSODs* genes, seven *SbPODs* genes, 11 sorghum lipoxygenase genes *SbLOXs*, and two *SbP5CS* genes, consistent with the physiological measurement of corresponding oxidative damage indicators: e.g., the enzyme activity of superoxide dismutase (SOD) and peroxidase (POD), and the content of malondialdehyde (MDA) and proline (Pro)).

Unlike the cold-stress response, sorghum exhibits substantially differing transcriptomic patterns in response to heat stress. According to one transcriptome study, the genes associated with high-temperature stress, intense light stress, and protein folding are differentially expressed during heat stress, with several genes encoding heat shock proteins or universal stress proteins—*HSP22.0* (Sb06g017850)*, HSP101* (Sb03g034390)*, HSP18.2* (Sb01g039990), and *SbUSP* (Sb04g034630)—being significantly upregulated (Johnson et al., 2014).

## Omics-enabled insights into plant responses to combinatory stresses

**Figure 1:**
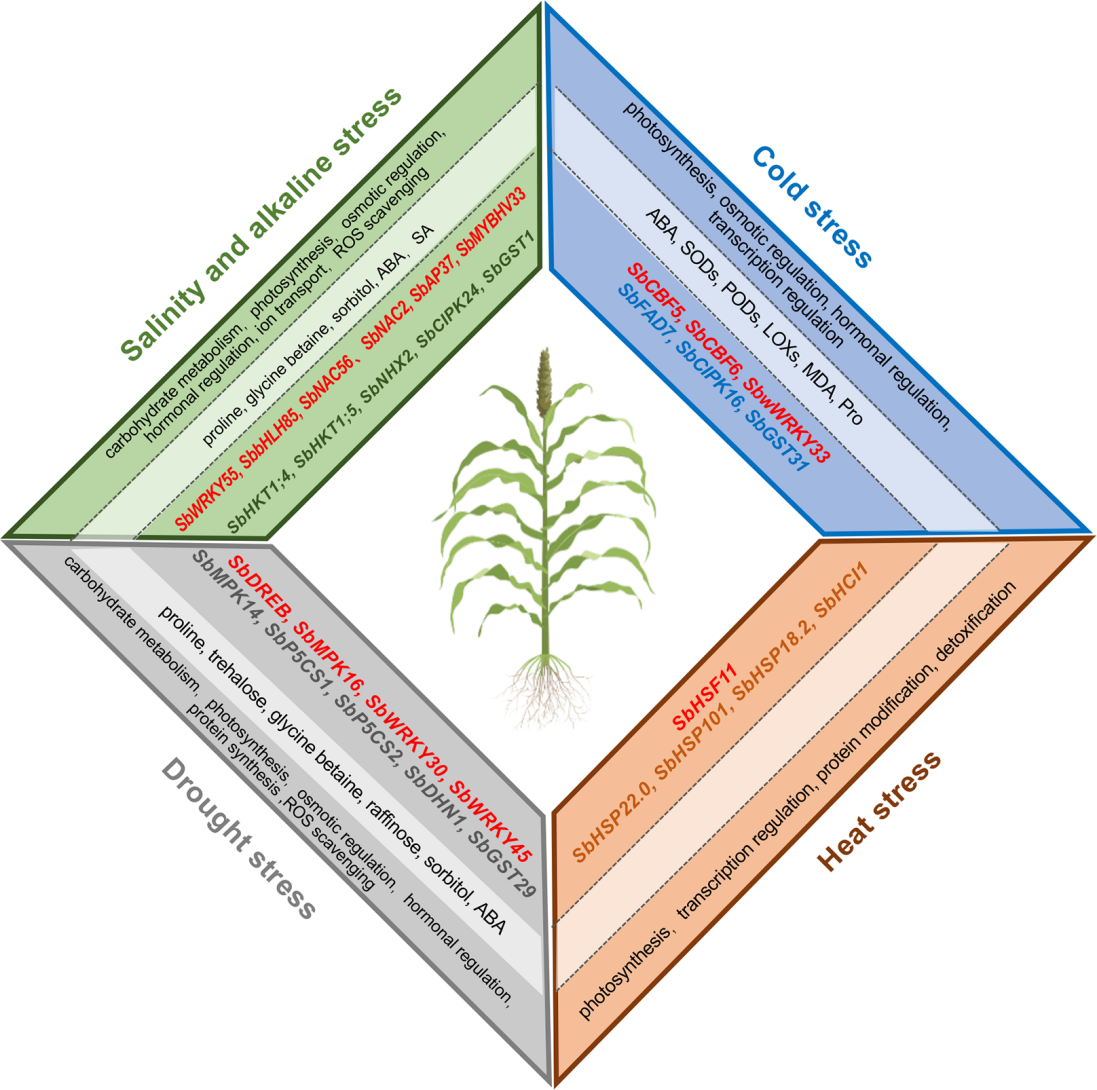
The diagram summarizing the molecular regulations involved in the response to and tolerance of abiotic stresses in sorghum. For each of the four abiotic stresses (drought, heat, cold, and salinity and alkaline) discussed herein, the candidate genes (transcription factors in red), metabolites, and physiological processes are depicted in three layers (from the innermost to outermost layer, respectively).

In the past decade, our understanding of the molecular responses to various stresses in sorghum has improved greatly, largely driven by advances and applications of the omics technologies. The major biological processes, metabolites (including phytohormones and osmolytes), and key genes that have been identified to respond to or cope with the abiotic stresses are summarized in **Figure 1**. Compiling and synthesizing these pieces of knowledge leads us to conclude that, in sorghum plants, different physiological and/or metabolic processes are affected by distinct stresses and are utilized to ensure survival and maintain growth when the stress occurs. For example, sorghum cultivar XGL-1 could induce the expression of proline biosynthetic and carbohydrate metabolic genes to cope with osmotic stress (Zhang et al., 2019), whereas the cultivar M-81E tends to amplify their gene expression of antioxidant enzymes and ion transporters when salt stress occurs (Yang et al., 2018). Moreover, imposed drought stress (withhold watering) and osmotic stress (with a PEG treatment) induce distinctive transcriptomic responses, with only ~20%-overlap of DEGs between the two treatments (Dugas et al., 2011; Johnson et al., 2014). Such pronounced differences in the stress-induced expression profiles have also been detected for heat and drought stresses. The drought treatment led to ~4% of those sorghum’s genes being differentially expressed, including those encoding the LEA proteins and proline synthetic enzyme P5CS2, while ~18% of them could be regulated in response to heat stress, including those encoding HSPs. By contrast, ~20% of the sorghum genes were DEGs under combined heat and drought stress, with around 1/3 of these DEGs being exclusively differentially expressed after applying the combined stresses (Johnson et al., 2014). These differences in stress-specific inducible genes in sorghum justify why it is imperative to compare the combination of stresses with each single stress at transcriptomic or other omic levels, given that such combinations typically arise in real field conditions. Therefore, the results may provide more practically meaningful insights into sorghum’s molecular breeding and stress tolerance improvement.

**Figure 2:**
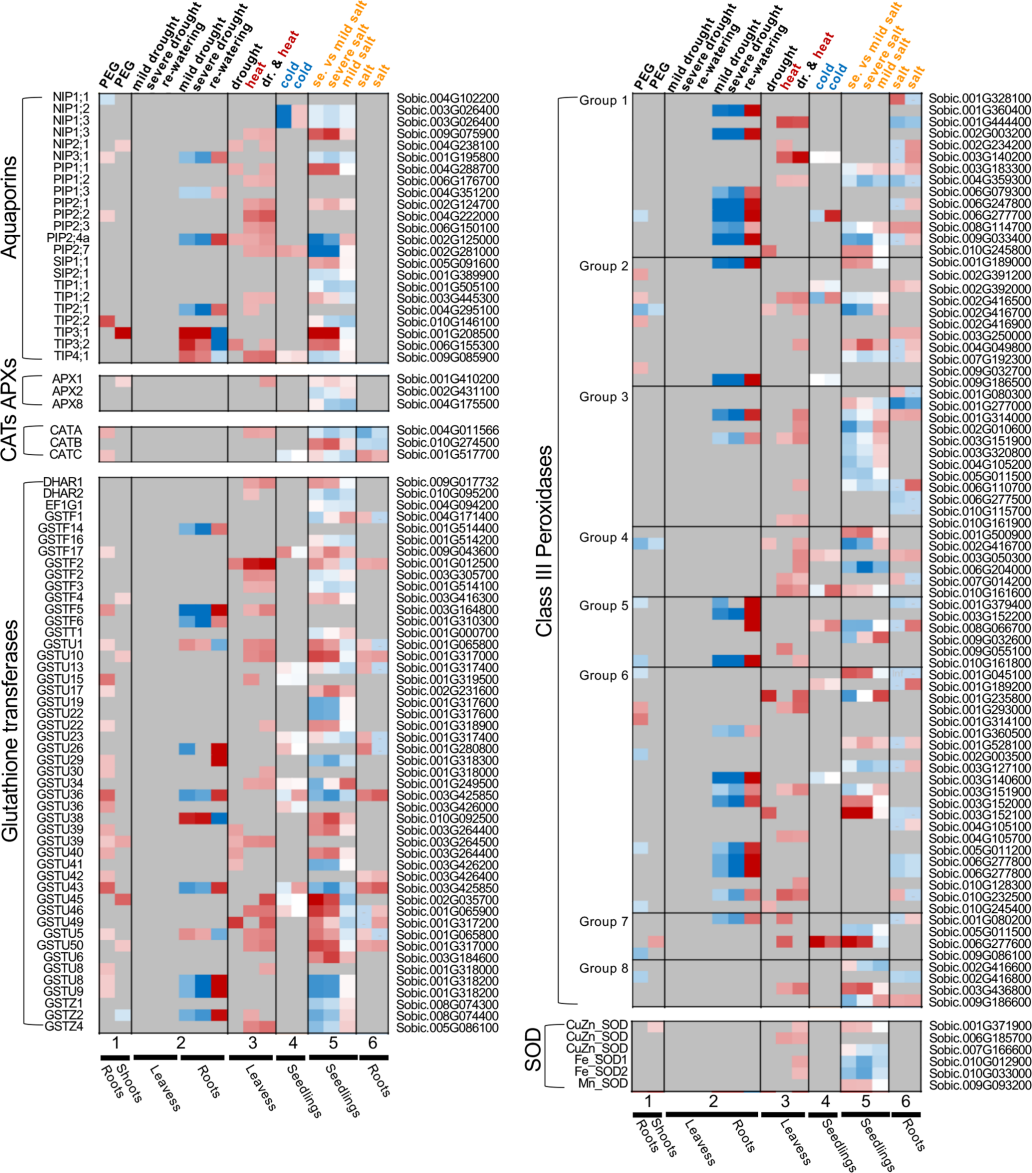
Comparison of the differences in gene expression in response to different abiotic stresses and their combination, exemplified using the stress-responsive gene families encoding aquaporins (AQPs) and the major antioxidant enzymes (i.e., APXs, CATs, GSTs, Prxs, and SODs). To avoid direct comparison of the expression levels between the studies, and to not include any batch effects between the studies, the log2(fold-change) of the expression data from each study were visualized by the heat map. These expression patterns are sorted by the studies (labeled as 1, 2, 3, 4, 5, 6 respectively for Dugas et al., 2011; Johnson et al., 2014; Chopra et al., 2015; Zhang et al., 2019; Azzouz-Olden et al., 2020; Ma et al., 2020, and Yang et al., 2018) and by the type of abiotic stress, with drought, heat, cold and salt stresses color-coded in black, red, blue and orange, respectively. Dr, drought. PEG, poly-ethylene glycol. For the study 2 (Dugas et al., 2011), fold changes of gene expression were compared between the leaf and root tissues; For the remaining studies, fold changes of gene expression were calculated in the way that the expression level in the non-stressed control as one. When a gene exhibited an increased or decreased expression (log2(fold-change)>1 or log2(fold-change)<-1), the color was indicated as red or blue, respectively, with the color shadiness indicating the extent of fold change.

The plant response at the physiological, gene expression, or protein level cannot be simply predicted by pooling existing knowledge acquired from single-stress treatment experiments. Such differences between the single stress and combined stress impacts and responses have been detected from the expression patterns of stress-responsive or ROS-scavenging genes in sorghum. A few studies on the changed patterns of gene expression under drought, heat/cold and salt stresses in sorghum are selected to visualize the distinct responses at the expression level (Dugas et al., 2011; Johnson et al., 2014; Chopra et al., 2015; Yang et al., 2018; Azzouz-Olden et al., 2020; Ma et al., 2020)—as the full list of DEGs are readily available from these papers or the **Supplemental Files**—to unbiasedly profile the ROS-scavenging genes. Aquaporin (*AQP*) genes were investigated as well because this gene family encodes the AQP membrane proteins which regulate membrane permeability to water and other molecules (Hu et al., 2012; Huang et al., 2014). The antioxidant system in plants includes several major enzymes (i.e., catalase, CAT, superoxide dismutase, SOD, class III peroxidase, CIII Prx, ascorbate peroxidase, APX, glutathione transferase, GST), which are encoded by corresponding gene families (Rajput et al., 2021). In **Figure 2**, certain genes are preferentially responsive to certain stress or respond in specific tissues. For example, most of the ROS-scavenging genes tend respond to the stresses in the root but not in the leaf. The *SbSODs* are only differentially expressed in the heat or salt stress samples. By contrast, tonoplast membrane intrinsic proteins (TIPs) from the *AQP* family are apt to be upregulated during drought stress conditions, while the plasma membrane intrinsic protein (PIP) subfamily responds to multiple stresses. Further, *APX* and *CAT* genes are differentially expressed under drought or salt stress. For the large antioxidant gene families, such as *GST* and *Prx*, their genes are responsive to the stress treatment in a member-specific pattern rather than a subfamily-specific pattern. The Class III Prxs catalyze H_2_O_2_, produce ROS (OH^-^ or O^2-^), and participate in diverse physiological processes, including seed germination, lignin metabolism, phytohormone catabolism, and pathogen resistance. Among the eight phylogenetic groups of Prxs (Liu et al., 2021), Prx groups 1, 5, and 6 tend to be responsive to drought stress, whereas Prx groups 2, 3, 7 and 8 tend to be responsive to salinity stress. The stark differences among the responsive patterns of antioxidant-related genes reflect well the different molecular regulations and specific ROS-scavenging capacity underlying the stresses.

On the other hand, different stresses could have the similar or distinct impacts on different physiological or biochemical pathways. For instance, in Arabidopsis, heat stress leads to elongated and thin leaves with increased leaf area and reduced root growth, while drought stress reduces leaf area and increases root growth to enhance water-use efficiency (Vile et al., 2012). However, both drought and heat stresses result in similar growth outcomes, including early flowering, higher rate of seed sterility, and yield losses (Zhang and Sonnewald, 2017). The counteracting effects of drought and heat stresses also depend on the stomata phenotype: heat stress leads to stomata opening to increase transpiration and to cool the leaf surface, while drought stress results in stomata closing to prevent water loss (Rizhsky et al., 2002; Rizhsky et al., 2004; Prasch and Sonnewald, 2015). By contrast, several stress treatments (i.e., drought, high salinity, or low temperature) all cause osmotic stress and oxidative damage (Mittler and Blumwald, 2010); to counteract the osmosis, sorghum plants generally secrete small molecules (such as soluble sugar, alginate, sorbitol, and proline) (Fang et al., 2021). These similarities and differences in physiological responses to various single stresses complicate our understanding of the molecular response to combined stresses.

Shaar-Moshe et al. (2017) investigated the transcriptional patterns and morpho-physiological acclimations of *Brachypodium distachyon* to single salinity, drought, and heat stresses, as well as their double- and triple-stress combinations. The combined stresses intensified the physiological effects when compared to single stresses, with some morphological traits more sensitive to salt stress while some physiological traits being more sensitive to heat stress. Transcriptome analysis revealed that the response patterns of the triple and the three double-stress combinations showed that only 37% of the common stress DEGs (574 genes out of 1550) maintained the same response mode, indicating limited consistency of expression for combined stresses. The response to heat stress at the transcriptional level contributed most to the major differential expression, with single and combined stresses with heat stress involvement showing stronger correlations. Conversely, single drought stress showed weaker correlations with both double- and triple-stress combinations with its involvement. Therefore, it is speculated that the contribution of drought in the combined stress treatments may lie with enhancing or attenuating the intensity of other stresses. In another example of the combination of drought and cold stresses that frequently occur in the northwestern and eastern China, Guo et al. (2021) investigated molecular responses of maize to the drought and cold combined stress. They showed that both single and combined stresses significantly reduced leaf photosynthesis. Nevertheless, the photosynthetic indicators were similar to the control plants in drought-treated and drought-and-cold-treated plants, whereas cold-treated plants were unable to recover during the recovery stage. Transcriptomic and metabolomic analysis further revealed that drought and cold interacted to mitigate this irreversible damage. This multi-omics study on combined stress in maize also provides the take home message that the outcome of combinatorial stress depends not only upon the nature of the involved stressors but is also related to developmental stages of the plant, the timing of stress applications, and the severity of individual stresses incurred. These factors contribute to the complexity and unpredictability of combinatorial stresses. Overall, these omics studies of single and combined stresses in Brachypodium and maize have provided direct evidence supporting that the molecular responses (at least at the gene expression level) to a combined stress could not be simply predicted in sorghum by the existing multi-omic-based knowledge obtained in single-stress conditions, justifying the importance to study crops’ response to combined stresses in agriculturally relevant circumstances.

## Concluding remarks and future perspective

Understanding the responses to abiotic stresses and the molecular regulation enacted to cope with stressful conditions faced by sorghum are of great importance. This is because not only does sorghum have strong tolerance to several major abiotic stresses, but it also is closely related to other agriculturally and economically important major crops (i.e., maize, sugarcane, and rice) with clear gene orthology/synteny between the species. Thus, our review does not only summarize a catalog of sorghum genes for molecular improvement but may also provide useful resource for stress-tolerance improvement in these related crops (**Table S1**).

The past decade has witnessed vast and rapid advances in our knowledge regarding the molecular responses of sorghum plants to drought, salinity-alkaline, and low/high temperature stresses. This burgeoning body of research has already pinpointed numerous pathways and genes that are associated with the tolerance of certain stress types and those should be prioritized for functional studies. Still, several challenges need to be addressed in future studies. (1) Arguably, systemic molecular insights into sorghum stress-tolerance can be obtained by integrating multi-omic technologies. Previous studies were dominated by transcriptomics with a few that used outdated proteomic technologies. Currently, proteomic technologies with higher throughput and sensitivity are available (Mergner et al., 2020), while metabolomics (such as widely-targeted metabolome) has become the mainstream tool to decipher the metabolite dynamics and genetic basis of plant stress response and regulation (Chen et al., 2013; Yuan et al., 2022). Single-cell omic and spatio-omic technologies have also been successfully applied in major crops (Do et al., 2016; Gutzat et al., 2020; Xu et al., 2020; Chen et al., 2021; Kajala et al., 2021; Li et al., 2021; Shaw et al., 2021; Wang Y. et al., 2021). The integration of multiple omics data and the application of cutting-edge technologies will broaden our understanding of the mechanisms related to stress response and regulation. (2) Large-scale comparisons between phenotypically diverse accessions would shed light on the genetic diversity and plasticity of stress tolerance in sorghum. Many previous studies relied on one or two cultivars at limited time points, thus we still lack large-scale comparisons across accessions and/or time series. (3) Combined stresses often happen in field conditions yet are heavily understudied in basic research. Clearly, our knowledge regarding the molecular response and resistance to combined stresses needs to be enhanced, but this usually cannot be simply inferred from existing knowledge obtained from single stress experiments. (4) The many candidate genes in sorghum revealed by omics are functionally known in other model species but they still lack functional validation in sorghum. Recently, sorghum’s transformation *via* particle bombardment and *Agrobacterium* has markedly improved with the greater use of morphogenesis genes and optimization of transformation details (Mookkan et al., 2017; Wu and Zhao, 2017; Kuriyama et al., 2019; Liu et al., 2019). These advances now pave the way for gene functional studies in sorghum and will surely provide direct support for molecular breeding efforts aimed at stress-tolerance improvement. In addition, with the rapidly accumulated omics data of sorghum’s abiotic stresses, the establishment of multi-omic platforms for sorghum and unified standards for its data aggregation and collation will greatly enhance both the mining and re-use of data for multi-omic research.

## Author contributions

Conceptualization, MT, CD, ZX and YL. Literature review, MT, CD, BY, GW, YD, YW, MC and YL. Drafting the original version of the manuscript, MT, CD and YL. Writing—review and editing, YW, MC, JC, GY, GH, ZX and YL. Supervision, GY, GH, ZX and YL. Funding acquisition, GY, GH, ZX and YL. All authors contributed to the article and approved the submitted version.
